# Poly-l-lysine-caused cell adhesion induces pyroptosis in THP-1 monocytes

**DOI:** 10.1515/biol-2022-0028

**Published:** 2022-03-24

**Authors:** Chaoping Yu, Wei Zhao, Chujun Duan, Jiangang Xie, Wen Yin

**Affiliations:** Department of Emergency, Xijing Hospital, Fourth Military Medical University, 127 West Changle Road, Xi’an, Shaanxi 710032, China

**Keywords:** pyroptosis, THP-1, monocytes, poly-l-lysine, cell adhesion

## Abstract

Pyroptosis is a kind of cell necrosis mediated by inflammasomes. The caspase 1-induced cleavage of gasdermin D (GSDMD) is a canonical pathway to cause membrane pores and eventually cell pyroptosis. Poly-l-lysine (PLL) is widely used to enhance cell adhesion during experiments. Human THP-1 cells are a typical cell line used to study pyroptosis due to their monocytic and macrophage-like characteristics. However, it was found that THP-1 cells seeded on the PLL-coated slides died. To figure out the reason, we observed the morphology of THP-1 cells on PLL-coated slides, which showed obvious pore forming on the cell membranes and cell swelling. The indicated pyroptosis-related protein expression was evaluated and it showed that the conventional caspase-1 pathway of pyroptosis was activated through the NLRP3 inflammasome in THP-1 monocytes on the PLL-coated slides. Hence, PLL-guided cell adhesion induces cell pyroptosis in THP-1 monocytes, which calls for THP-1 dominant studies of pyroptosis to avoid the use of PLL-coated slides or PLL-related drugs.

## 1 Introduction

Pyroptosis is generally defined as a programmed cell death pathway triggered by inflammasomes, which is then mediated by pore-forming gasdermin family members [1,2]. Since the term “pyroptosis” was proposed in 2001 [3], the mechanism of this programmed cell death has been extensively studied. In the canonical pathway of pyroptosis, activation of inflammatory caspase 1 through various inflammasomes, like NOD-like receptors (NLRs), drives the cleavage of gasdermin D (GSDMD) [4]. The released N-terminal domain of GSDMD (GSDMD-NT) promotes the formation of membrane pores and also the release of specific interleukins (IL-1β and IL-18), leading to cell death [5,6]. It was found that apart from the traditional caspase 1-mediated cell death, other caspases, including inflammatory caspases 4/5/11 and apoptotic caspase 3, were also involved in the process of gasdermin cleavage to trigger cell pyroptosis [7,8,9]. Moreover, studies have revealed that pyroptosis is engaged in the progression of multiple diseases, especially in cancers, atherosclerosis, and some inflammatory disorders [10,11,12]. Although the mechanism of cellular pyroptosis has been gradually clarified, further investigations of its molecular interactions were also necessary to be studied in various pathological models. Therefore, it is of great experimental and clinical significance to further explore the occurrence and application of cell pyroptosis.

Cell adhesion is an important issue to be mastered in cytological research. Poly-l-lysine (PLL)-coated glass slides are a traditional way to increase the adhesion capability of cells [13,14]. As a coating material, PLL is a cationic polymerized amino acid that can attract eukaryotic cells or bacteria through electrostatic binding effects [15,16,17]. Besides, the human THP-1 cells are a monocytic leukemia cell line derived from the blood sample of a young male patient with acute monocytic leukemia [18]. As a suspension cell line, THP-1 can not only retain the function of monocytes but also differentiate into macrophage-like cells through the induction of phorbol ester (PMA) and release cytokines [18,19,20]. Because of these characteristics, THP-1 cells have been widely used to explore the physiological and pathological processes of immune and inflammation-related disorders *in vitro*, in particular pyroptosis [20,21,22,23]. Although PLL is a common method to decorate slides, we found in this study that THP-1 cells binding to PLL-coated slides easily caused cell pyroptosis, leading to cell rupture and death; this phenomenon is worth mentioning and of concern to avoid possible risks in further related studies.

## 2 Methods and materials

### 2.1 Cell culture and electron microscopy

Human THP-1 monocytes were purchased from ATCC (USA). The cells were maintained in the RPMI-1640 medium (Corning, USA) supplemented with 10% fetal bovine serum (FBS), 100 U/mL penicillin, and 100 μg/mL streptomycin (Corning) at 37°C, with 5% CO_2_. The cells were divided into three groups: control, slide only (the cells were added to the slides without any treatment), and PLL-coated slide (the cells were placed on the slides coated with PLL). Before cell attachment, the glass slides were soaked overnight in a diluted PLL solution (#P4832, molecular weight: 150,000–300,000, Sigma-Aldrich, Germany) with a final concentration of 0.01 mg/mL, followed by washing 3 times with phosphate-buffered saline (PBS). The reagent is sterile and LPS contamination-free. After the addition of THP-1 cells onto the slides and adhesion for 12 h, the samples were observed and captured under a scanning electron microscope (SEM, #S-4800, Hitachi, Japan) with ×10,000 or ×30,000 magnification.

### 2.2 Western blotting

After adhesion, the total protein samples from the THP-1 cells were extracted with the RIPA lysis buffer (GenStar, China). BCA protein assay kit (GenStar) was adopted to measure the protein concentration. SDS-PAGE was performed with each lane containing 20 μg of protein samples. Separated proteins were transferred onto the PVDF membranes (Merck Millipore, Germany), followed by blocking in 5% non-fat milk powder and washing with TBST. The membranes were incubated overnight with diluted (1:1,000) primary antibodies, including anti-caspase 1 (#ab179515, Abcam, China), anti-cleaved-GSDMD (#36425, Cell Signaling Technology (CST), China), anti-NLRP3 (#15101, CST), and anti-β-actin (#C4, Diyibio, China), at 4°C. HRP-labeled anti-rabbit and anti-mouse IgG secondary antibodies (CST) were used for subsequent incubation of the membranes at room temperature for 1 h. The enhanced chemiluminescence (ECL) solution (Fdbio Science, China) was added to the membranes, followed by detection through a chemiluminescence imaging system (Baygene, China).

### 2.3 Statistical analysis

Data analysis between the three groups was carried out using ordinary one-way ANOVA, combined with a Tukey multiple comparison test with GraphPad Prism 8 (GraphPad Software, USA). Statistical significance was defined when *P* < 0.05.

## 3 Results

**Figure 1:**
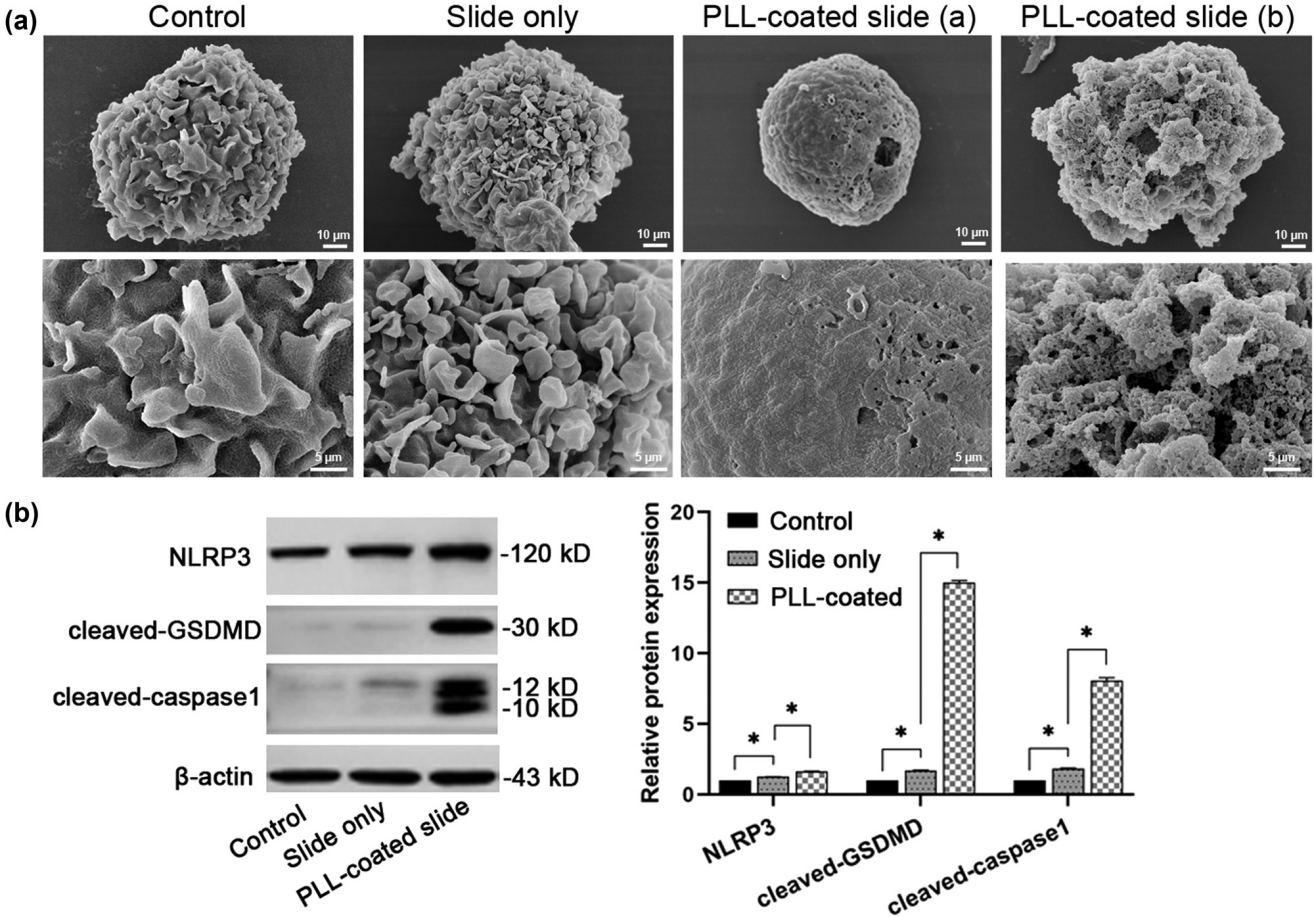
PLL-caused cell adhesion induced pyroptosis in THP-1 monocytes. (a) Morphology of THP-1 cells. SEM images of the cells in the control, slide only, and PLL-coated slide groups (from left to right). Upper scale bar: 10 μm; lower scale bar: 5 μm. (b) Western blotting images and quantification of indicated pyroptotic proteins (NLRP3, cleaved-GSDMD, and cleaved-caspase 1) in THP-1 cells in different groups. *P < 0.05; *N* = 3.

The morphology of THP-1 monocytes on the normal and PLL-coated glass slides was detected using an SEM. It was shown that the cells in the control and slide only groups had normal cell morphology with natural size and intact membranes. However, THP-1 cells on the PLL-coated slides exhibited incomplete cell membranes (obvious pore-forming) combined with (a) cell swelling and subsequent (b) membrane rupture (Figure 1a), all of which were the typical structural features of pyroptosis [2], suggesting that pyroptosis may occur in THP-1 monocytes under this situation. To verify our speculation, the relative protein expression of specific pyroptotic indicators in the THP-1 cells after adhesion was evaluated using western blotting, which showed that the cells on the PLL-coated slides contained significantly increased protein expression levels of NLRP3, cleaved-GSDMD (GSDMD-NT), and cleaved caspase 1, compared to the controls (Figure 1b). Therefore, our findings indicate that PLL-caused cell adhesion induces cell pyroptosis in THP-1 monocytes through activation of the caspase 1/GSDMD pathway via the NLRP3 inflammasome. Although it is common to use PLL-coated slides to enhance cell adhesion and minimize cell loss during cellular experiments [14], this method may be unsuitable for THP-1 cells in pyroptosis-related research.

## 4 Discussion

As extensive applications of THP-1 cells in the study of cell pyroptosis, increasing factors and effects have been reported to induce this process. Among them, lipopolysaccharide (LPS) is the first reported canonical factor to induce cell pyroptosis in eukaryotes, including THP-1 monocytes [7,22]. A recent study found that dipeptidyl peptidase inhibitor Val-boroPro (VbP) induced pyroptosis in THP-1 macrophages, which could be inhibited by electrical stimulation [24]. The thymic stromal lymphopoietin (TSLP) in fungal keratitis and the infection of Middle East respiratory syndrome coronavirus (MERS-CoV) also showed similar effects to induce pyroptosis of THP-1 macrophages [23,25]. From our observation, it seems that PLL may also have the ability to induce pyroptosis in THP-1 monocytes, which provides a novel perspective to study the pyroptotic process of human monocytes.

THP-1 cells are a typical human cell line cultured in suspension. Rodig has reported that PLL, due to its charged side chain, is strongly recommended for the attachment of suspension cells to conduct cell staining [26], but this operation was not applicable to THP-1 cells in our study. Nevertheless, the underlying mechanism of the activation of pyroptosis in THP-1 cells induced by PLL-coated slides was still unclear. Previous findings indicated that the molecular weight and the concentration of PLL were positively correlated with its adhesive force [15]. Symonds et al. found that PLLs with low and high molecular weights can induce distinct mitochondrial-dependent cell apoptosis in different human cells [27]. In addition, PLL has been recognized as a polymer with relatively high cytotoxicity, which was also confirmed to be related to the treating time and concentration [28]. Since apoptosis is also accompanied by alterations of membrane permeability [29], we cannot deny the occurrence of cell apoptosis and the changes of other apoptosis-related caspases and gasdermins (such as caspase 3-regulated GSDME activation [30]) in the current study, which needs further exploration. However, the obvious canonical pyroptosis features of THP-1 cells at least suggested that pyroptosis is the primary mechanism to cause THP-1 cell death on the PLL-coated slides here.

On the other hand, since THP-1 monocytes were a suspension cell line isolated from peripheral blood [31], it is speculated that the passive deformation of the cells would more or less hinder their normal cell functions and bring negative effects on themselves. Another research indicated that asbestos and silica could stimulate NLRP3-dominant secretion of IL-1β in THP-1 macrophages, which may trigger pulmonary fibrosis and diseases [32]. Pneumoconiosis induced by inhalation of silica and silicosis by long-term silica exposure is well-known to be regulated by the activation of NLRP3 inflammasome [33,34]. As NLRP3 inflammasome activation is a pivotal step to cause caspase 1-mediated release of proinflammatory cytokines and GSDMD-dominant pyroptosis [35], the abovementioned literature also provides supportive evidence for our observations from the slide material. Hence, the cytotoxicity of PLL, silicate material of glass slices, and the immobilization of THP-1 monocytes might be crucial stimuli to induce the activation of caspase 1-mediated pyroptotic pathway in THP-1 cells on PLL slides, causing cell death eventually. Follow-up experiments should be considered to further verify these factors; however, according to the current study, we suggest that when THP-1 monocytes are used as a major study object *in vitro*, researchers should pay more attention to avoid using PLL as a coating material to enhance cell adhesion, thereby preventing unnecessary pyroptosis activation and cell death.

Nowadays, other cell adhesive materials have also been reported for specific cell types. Research indicated that fibronectin and collagen-modified cultural scaffold promoted cell attachment of adipose-derived stromal cells [36]. Surface-modified polyvinylidene fluoride with fibronectin binding also showed enhanced cell attachment in osteoblast [37]. However, their adhesion effects on THP-1 monocytes still need to be further studied. In addition, a protocol for suspension cell adhesion, including THP-1 cells, using serum-free conditions has been presented [38], which could be considered to replace the PLL-coated slides. Nevertheless, the improvement of the experimental methods to promote suspension cell attachment while maintaining the integrity of the cell structure and functions remains an issue that needs further exploration.
